# Modulating Ferroptosis: A Novel Approach to Promote Neural Repair in Brain Injury

**DOI:** 10.2174/011570159X343096241209040135

**Published:** 2025-01-15

**Authors:** Yanlin Liu, Ying Guo, Huixin Zhou, Yuxin Liu, Yuting Jin, Kaitao Luo, Xiaobing Dou

**Affiliations:** 1 College of Life Sciences, Zhejiang Chinese Medical University, Hangzhou, Zhejiang 310053, China;; 2 Department of Acupuncture and Moxibustion, Jiaxing Hospital of Traditional Chinese Medicine, Jiaxing, Zhejiang 314033, China;; 3 Department of Acupuncture and Moxibustion, The Second Affiliated Hospital of Heilongjiang University of Traditional Chinese Medicine, Harbin, Heilongjiang 150001, China;; 4 Department of Rehabilitation, Jiaxing Hospital of Traditional Chinese Medicine, Jiaxing, Zhejiang 314033, China;; 5 Third Clinical Medical College, Zhejiang Chinese Medical University, Hangzhou, Zhejiang 310053, China

**Keywords:** Brain injury, nerve repair, ferroptosis, inflammation, ferroptosis inhibitors, neurodegeneration

## Abstract

The increasing prevalence of brain injuries resulting in cognitive and motor function impairments poses a substantial global medical challenge. Nerve repair therapies offer promise for addressing brain injury-related disorders. Ferroptosis, as a cell death mechanism associated with oxidative stress and inflammation. Certain ferroptosis inhibitors, such as iron chelators and antioxidants, exhibit therapeutic potential for brain injury-related conditions. This review explores the fundamental processes and associated mechanisms that regulate neural repair by inhibiting ferroptosis, thereby alleviating brain injury and promoting neuroregeneration. Furthermore, it examines the action mechanisms and potential therapeutic applications of ferroptosis inhibitors in neural repair, aiming to provide novel insights for treating brain injuries.

## 1 INTRODUCTION

The global prevalence of brain injury is increasing, primarily attributed to demographic aging and a surge in accidental occurrences [1, 2]. Brain injury can result in persistent physical and cognitive deficits, including movement disorders, memory impairment, emotional volatility, social dysfunction, and diminished quality of life, which impose a substantial burden on both individuals affected by the injury and their families [3, 4]. Neuronal injury represents a significant form of brain injury, originating from various factors such as external impact, ischemia, infection, toxin exposure, or disease progression. These injuries can lead to neuronal loss, disrupted synaptic connections, and hindered neuronal network reorganization, affecting brain function [5, 6]. Traditional methods for treating brain injury include pharmacological treatment, surgical intervention, and stem cell therapy [7]. Specifically, neuroprotective agents such as NMDA receptor antagonists help reduce neural damage and cell death [8], while anti-inflammatory drugs like corticosteroids (*e.g*., dexamethasone) mitigate inflammation in brain tissue [9]. Surgical interventions, such as craniectomy, effectively relieve severe intracranial pressure elevation [10]. Stem cell therapy, involving the use of autologous or allogeneic stem cells, promotes neural regeneration [11]. Additionally, recent research has begun to explore novel therapeutic strategies that integrate traditional medicine with modern biomedical science. Traditional Chinese medicine (TCM)-based approaches, which utilize herbal compounds known for their neuroprotective properties, offer new potential avenues for the treatment of brain injuries [12]. However, the existing treatments have limitations in managing severe or specific types of brain injuries, highlighting the need for the development of new therapeutic strategies.

While nerve tissue is considered to have relatively limited regenerative capacity, particularly in the central nervous system such as the brain and spinal cord, it may still be possible to promote the regeneration and repair of nerve tissue through specialized approaches following severe nerve damage [13, 14]. Nerve repair is a complex but promising area of study, with the focus centered on balancing the signaling pathways between cell survival and cell death [15, 16]. Apoptosis, autophagy, and necrosis are traditional modes of cell death [17, 18]. Ferroptosis, a form of programmed cell death significantly differs from traditional pathways of cell death, distinguished by its specific molecular mechanisms and biochemical characteristics [19]. The main characteristics of ferroptosis include the accumulation of intracellular iron ions and the extensive production of lipid peroxides. This oxidative process primarily impacts the polyunsaturated fatty acids of cellular membranes, resulting in structural damage and ultimately initiating cell demise [20]. Prior research has indicated a potential correlation between ferroptosis and the balance of neuronal survival and death during nerve regeneration following brain injury. In cases of nerve injury, the inflammatory reaction may intensify cellular oxidative stress and lipid peroxidation, consequently triggering or expediting ferroptosis. However, strategic modulation of ferroptosis could potentially mitigate neuronal loss, facilitate neuronal regeneration, and enhance synaptic reconstruction, thereby enhancing the efficacy of nerve repair procedures [21, 22]. Studies have shown that ferroptosis plays a significant role in the pathophysiology of traumatic brain injury (TBI). Netrin-1 (Netrin-1) exerts neuroprotective effects by activating the UNC5B/Nrf2 signaling pathway, which promotes GPX4 expression, thereby reducing brain injury [23]. Cao *et al*. found that the ferroptosis inhibitor Liproxstatin-1 significantly alleviated neuronal damage and inflammatory response following subarachnoid hemorrhage (SAH) by inhibiting ferroptosis and reducing neuroinflammation [24]. Therefore, regulating the mechanisms of ferroptosis may offer new therapeutic avenues for neural repair.

Although the role of ferroptosis in neurodegenerative diseases has been extensively studied, its implications in neural repair following brain injury remain underexplored. This review provides a comprehensive analysis of the mechanisms underlying ferroptosis following brain injury and the regulatory processes that facilitate neural repair through ferroptosis inhibition. It systematically links ferroptosis regulation with brain injury repair. Additionally, we explore the potential and application of ferroptosis inhibitors, including herbal compounds from TCM herbs in neural repair, offering significant insights for the development of novel therapeutic strategies.

## 2 FERROPTOSIS AND BRAIN INJURY

### 2.1 The Mechanism of Ferroptosis

In various human body tissues and cells, including neurons, transferrin (TF) binds to transferrin receptor 1 (TFR1) to facilitate the cellular uptake of free ferrous iron (Fe^2+^) and iron (Fe^3+^), forming TF-Fe^3+^ complexes. Subsequently, prostate 6 transmembrane protein 3 (STEAP3) promotes the release of Fe^3+^ from TF and its conversion to Fe^2+^ [25]. Fe^2+^ is the bioactive form of iron that is more readily utilized by cells. Excess iron can be transported out of cells *via* ferroportin (FPN) or stored in ferritin. Additionally, iron can be released into the labile iron pool (LIP) from endosomes through the action of divalent metal transporter 1 (DMT1) to prevent iron overload [26]. These mechanisms collectively function to prevent iron overload. However, when these regulatory processes are compromised or when iron intake exceeds capacity, it can result in pathological elevations in systemic iron levels. This imbalance can induce intracellular lipid peroxidation and inflammation responses, thereby promoting ferroptosis [27].

### 2.2 Types of Brain Injury

Brain injury refers to damage to the structure of the brain caused by external forces or certain pathological changes [28]. Brain injury can be broadly categorized into two main types: TBI and non-traumatic brain injury (nTBI). TBI is defined as brain damage resulting from external impact, often stemming from events such as vehicular collisions, falls, and assaults. TBI can be further subdivided into mild TBI, commonly referred to as concussion, with symptoms including transient loss of consciousness, headaches, dizziness, *etc*.; moderate and severe TBI, which may result in prolonged loss of consciousness, memory loss, cognitive impairments, and other more serious consequences [1, 29]. nTBI can be caused by various internal medical reasons, among which conditions such as stroke, cerebral infarction, and intracerebral hemorrhage can lead to restricted or interrupted blood flow to the brain [30]. Due to the increased demand for oxygen and nutrients by brain tissue cells, interruptions in blood supply can impair energy metabolism in nerve cells [31]. Additionally, neurological disorders such as encephalitis and brain tumors can also cause severe damage to the cells [32, 33].

### 2.3 Accumulation of Iron Ions in Brain Injury

**Fig. (1):**
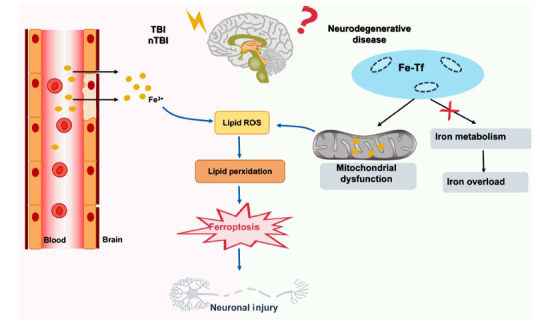
Iron ion accumulation and neuronal ferroptosis induced by brain injury.

The accumulation of iron ions resulting from brain injury promotes the occurrence of neuronal ferroptosis. In brain injury, iron ions can accumulate due to various complex mechanisms. For example, in TBI, iron ions from the blood may infiltrate brain tissue through damaged blood vessel walls or the blood-brain barrier, leading to accumulation [34]. Similarly, in nTBI, impaired blood-brain barrier function can also cause iron ion accumulation [35]. Furthermore, in neurodegenerative diseases, abnormal iron ion metabolism results in aberrant intracellular distribution of iron ions. Neurodegenerative diseases are also accompanied by declining mitochondrial function and increased oxidative stress, leading to abnormal intracellular iron ion accumulation. These factors collectively contribute to abnormal iron ion accumulation in the nervous system, exacerbating the occurrence and progression of neurodegenerative diseases [36, 37] (Fig. **1**).

### 2.4 The Role of Ferroptosis in Clinical Assessment and Post-mortem Analysis of Brain Injury

Given the occurrence of ferroptosis following brain injury, this phenomenon has crucial implications for clinical practice. By identifying molecular markers of ferroptosis, clinicians can assess the severity of brain injury earlier and more accurately, enabling the development of personalized treatment plans [38]. Furthermore, in post-mortem studies, the detection of ferroptosis-related markers in autopsy samples could aid in enhancing the understanding of the pathological processes involved in brain injury [39, 40]. While ferroptosis alone may not be sufficient to independently determine the type of brain injury or establish the exact cause of death during post-mortem examinations, it can be combined with other pathological, molecular, and forensic evidence to help elucidate the mechanisms of death and underlying pathological processes.

### 2.5 The basic Processes and Mechanisms of Nerve Repair

Neural repair is a process that promotes the regeneration and restoration of impaired neurons through a series of cellular and molecular mechanisms [41, 42]. When the nervous system is damaged, alterations in cell membrane integrity and membrane potential activate signal transduction pathways, triggering cellular stress responses and the release of inflammatory factors. This leads to infiltration of inflammatory cells and increased secretion of inflammatory mediators, establishing a positive feedback loop of the inflammatory response to clear debris and dead cells from damaged tissues, marking the onset of the neural repair process [43, 44].

Simultaneously, stem cells and precursor cells in the nervous system become activated and participate in neural regeneration and repair, differentiating into neurons, astrocytes, and other cell types to fill damaged areas and promote the regeneration and repair of impaired nerve tissue [45]. During neural regeneration, newly generated neurons begin to grow in the damaged area, attempting to re-establish a functional neural network, including axonal and dendritic development, synaptogenesis, and establishment of connections between neurons. As new neurons extend and synapses develop, the composition and operation of the damaged nerve tissue gradually repair, striving to restore typical functionality to the affected area [46, 47] Fig. (**2**).

### 2.6 Effect of Ferroptosis on Nerve Repair

Neural stem cells are a group of cells characterized by their ability to self-renew and differentiate into various cell types, contributing significantly to the restoration and rejuvenation of the nervous system [48]. During the nerve repair following brain injury, neural stem cells can undergo differentiation into various types of nerve cells and contribute to the development of novel neural networks [49]. Nevertheless, ferroptosis could potentially influence the functionality and destiny of neural stem cells. Research indicates that ferroptosis can directly result in the demise of neural stem cells, especially under conditions of elevated iron ion levels or prolonged exposure [50]. Iron overload can trigger oxidative stress, damaging crucial biomolecules within cells, ultimately leading to cellular dysfunction and apoptosis [51]. Additionally, ferroptosis may interfere with the proliferation and differentiation of neural stem cells, reducing their involvement in neural repair processes [52, 53].

**Fig. (3):**
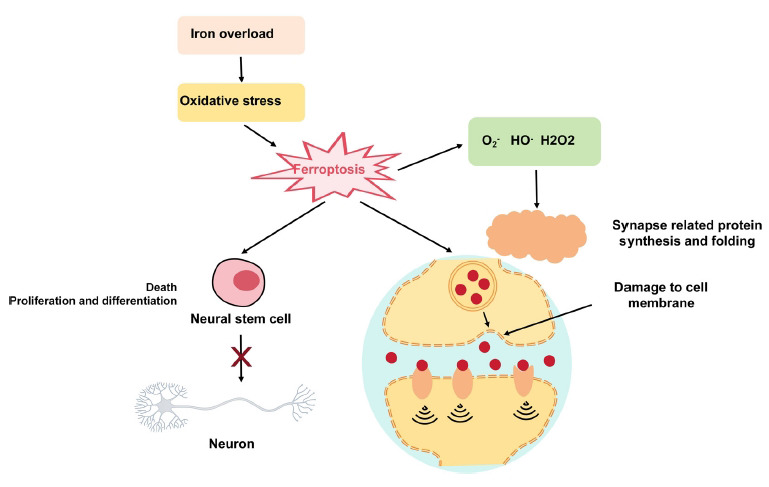
Effect of ferroptosis on nerve repair.

Synapses play a crucial role in the nervous system by facilitating the transmission of information between neurons, making them essential for the functionality and plasticity of the nervous system. During the process of nerve regeneration, the restoration of synapses is a pivotal stage in the restoration of neuronal function [54]. However, ferroptosis impedes the formation of synapses, thereby affecting the plasticity of the nervous system. The process of synapse formation necessitates cellular membrane interactions among neurons, involving neurotransmitter release from presynaptic neurons and subsequent binding to the cell membranes of postsynaptic neurons. Research indicates that the demise of nerve cells induced by Ferroptosis results in the disruption of synaptic networks, thereby impacting regular inter-neuronal communication [55]. Furthermore, the excessive buildup of lipid peroxide on the cell membrane resulting from Ferroptosis disrupts the structural integrity of the cell membrane and impedes the formation of synapses [56]. Moreover, the process of synaptic formation necessitates meticulous control over numerous proteins and signaling molecules. Oxidative stress leads to elevated levels of oxygen free radicals and other detrimental oxidative compounds, disrupting the intracellular environment and impeding the synthesis and folding of proteins associated with synapses [57, 58]. Therefore, regulating iron metabolism and reducing the accumulation of iron ions may be crucial strategies for promoting neural repair (Fig. **3**).

## 3 MOLECULAR MECHANISMS OF FERROPTOSIS REGULATION AFTER BRAIN INJURY

### 3.1 Interaction of Ferroptosis and Oxidative Stress

After brain injury, ferroptosis and oxidative stress are two key pathological processes. They interact with each other, exacerbating damage to brain tissue and functional impairment [59]. The key step in ferroptosis is lipid peroxidation, a process dependent on the generation of reactive oxygen species (ROS). After brain injury, the increased generation of ROS under oxidative stress conditions directly promotes lipid peroxidation [60]. Furthermore, ROS also facilitates the release of free iron ions by oxidizing ferritin, elevating intracellular iron ion levels, thereby accelerating the occurrence of ferroptosis [61]. On the other hand, iron ions can serve as cofactors in regulating oxidative stress signaling pathways. For instance, iron ions can influence the Nrf2-ARE signaling pathway, which regulates the expression of genes involved in the cellular antioxidant defense system to cope with oxidative stress [62]. In summary, oxidative stress and ferroptosis can mutually reinforce each other, exacerbating damage and death of neuronal cells.

### 3.2 Interaction of Ferroptosis with Inflammatory Responses

The inflammatory response following brain injury is triggered by the brain tissue's defense and repair mechanisms in response to the damage. Following brain injury, ferroptosis and the inflammatory response interact through various mechanisms. Firstly, ferroptosis can promote the inflammatory response through the following pathways: (1) Ferroptosis leads to cell membrane rupture, releasing cellular contents such as DAMPs (damage-associated molecular patterns), which activate inflammatory cells and responses [63]; (2) Lipid peroxides and other ROS serve as inflammatory signaling molecules, activating microglia and macrophages, thereby promoting the release of inflammatory factors [64]. On the other hand, the inflammatory response also exacerbates ferroptosis by regulating the accumulation of iron ions and oxidative stress levels [65].

## 4 RESEARCH PROGRESS AND CLINICAL APPLICATION PROSPECTS OF FERROPTOSIS INHIBITORS

### 4.1 The Action Mechanism of Ferroptosis Inhibitors

Ferroptosis inhibitors hold substantial importance in facilitating neural recovery following brain injury. These inhibitors function by modulating the buildup of iron ions and oxidative stress levels to impede ferroptosis, consequently safeguarding the viability and functionality of nerve cells. Categorized based on their mode of operation and chemical composition, ferroptosis inhibitors can be classified into distinct groups including iron chelators, redox regulators, and antioxidants [66]. Iron chelators exhibit a strong attraction to iron ions, enabling the formation of durable complexes that effectively deactivate iron ions. This process helps mitigate oxidative stress and lipid peroxidation. Iron chelators such as Desferrioxamine (DFO) and Deferiprone (DFP), both of which contain iron complex groups with a high affinity for iron ions [67, 68]. Redox regulators play a crucial role in safeguarding cells against oxygen radical-induced harm by maintaining the redox equilibrium, whereas antioxidants mitigate oxidative stress, exemplified by compounds like N-acetylcysteine (NAC) and Edaravone, primarily through the scavenging of free radicals [69, 70]. Iron channel blockers like Deferasirox work to decrease the buildup of iron within cells by obstructing iron channels located on cell membranes, thereby impeding the entry of external iron ions into the cell [71]. Ferritin enhancers stimulate the process of iron ion storage and transportation by boosting ferritin production and functionality, thereby elevating its intracellular levels [72].

### 4.2 Therapeutic effect of Ferroptosis Inhibitors

Zhu *et al*. validated the therapeutic effects of ferroptosis inhibitors in Alzheimer's disease (AD) using a mouse model. They found administration of DFO effectively alleviates symptoms of AD in mice. This is because DFO chelates iron ions, reducing levels of iron ions and ROS, thereby inhibiting lipid peroxidation and oxidative stress, and protecting neurons from damage caused by ferroptosis [73]. It has also been found to mitigate neuroinflammatory harm in cells by suppressing ferroptosis [74]. Sripetchiwandi *et al*. noted that the administration of DFP in combination with a specific calcium channel blocker, efonidipine, resulted in the mitigation of neurotoxic effects induced by thalassemia [75]. Zheng *et al*. noted that α-lipoic acid (ALA) functions as an antioxidant and iron chelator, regulating iron metabolism by increasing the expression of iron FPN and decreasing the expression of iron uptake transporter DMT1 in models of Parkinson's disease (PD). Additionally, ALA was found to protect against mitochondrial damage in these models by reducing the buildup of ROS and lipid peroxides through the inhibition of glutathione peroxidase 4 (GPX4) and cysteine/glutamate transporter (xCT) downregulation [76]. Lee and colleagues discovered that PPARδ agonists, specifically GW501516, can reduce the neurotoxic effects induced by 6-hydroxydopamine (6-OHDA) through inhibiting intracellular iron buildup. This inhibition decreases the production of ROS and lipid peroxides linked to iron overload, consequently contributing to the amelioration of PD [77].

### 4.3 Potential Novel Ferroptosis Inhibitors

Ferroptosis inhibitors demonstrated significant potential in treating brain injuries and promoting neural regeneration, with important implications and application prospects for both single-target and multi-target interventions. Single-target interventions offer high specificity and fewer side effects, making them suitable for the precise treatment of specific diseases. However, given the complex etiologies of conditions like AD, PD, and ischemic brain injury, achieving optimal therapeutic outcomes through single-target interventions can be challenging. Multi-target interventions provide more comprehensive and effective protection by simultaneously targeting multiple ferroptosis pathways. A typical multi-target intervention agent, niacin dimer (N2L), has been found to regulate oxidative stress and iron metabolism through multiple pathways, thereby inhibiting lipid peroxidation and ferroptosis, which exhibits potential as a ferroptosis inhibitor and may play a role in the treatment of neurodegenerative diseases associated with ferroptosis [78]. Tetrahydroxystilbene glycosides (TSG) improve neural function in brain injury models by inhibiting ferroptosis and reducing oxidative stress through the stimulation of the GSH GPX4 signaling pathway. Although its clinical application is still under investigation, the neuroprotective effects of TSG make it a promising candidate for the treatment of brain injury [79]. The compound CMS121, a derivative of fisetin, can eliminate intermediate peroxides, specifically 4-HNE, and mitigate cognitive deterioration [80].

Furthermore, TCM approaches also show unique advantages in inhibiting ferroptosis [81]. Data from Table **1** indicate that certain natural plant extracts show significant effects in managing brain injury-related conditions by modulating ferroptosis levels. These substances exhibit multi-target characteristics. For example, the study found that Rehmannioside A can improve cognitive dysfunction following ischemia and alleviate ferroptosis by activating the PI3K/AKT/Nrf2 and SLC7A11/GPX4 signaling pathways. Moreover, Rehmannioside A treatment resulted in significant cognitive function improvement in animal models, suggesting that DHM may be a potential neuroprotective agent [82]. Puerarin is a natural flavonoid extracted from kudzu root, known for its antioxidant, anti-inflammatory, and neuroprotective properties. It is therefore considered a potential agent in addressing iron-related oxidative damage [83]. Zhang *et al*. explored the protective effects of Puerarin in brain ischemia-reperfusion injury, particularly focusing on its ability to regulate the PI3K/Akt/Nrf2 signaling pathway to inhibit oxidative stress and alleviate damage [84]. These studies suggest that plant-derived compounds have potential as novel ferroptosis inhibitors in the management of brain injury and promotion of neural regeneration. However, their effectiveness and methodologies in clinical applications require further exploration and evaluation.

In terms of diagnosis and treatment, theranostic microRNA (miRNAs) also show promising applications. Specifically, Li *et al*. investigated the expression patterns of specific miRNAs in AD and alcohol dependence and explored their association with changes in ferroptosis-related gene expression. Through gene-miRNA interaction network analysis, it was found that hsa-miR-34a-5p and hsa-miR-106b-5p simultaneously regulate the expression of key ferroptosis-related genes (CYBB and ACSL4), making them not only serve as disease regulatory factors but also have the potential to become diagnostic biomarkers for AD and alcohol dependence [85]. Additionally, in patients with AD and preclinical AD (PAD), plasma levels of miR-34a-5p and miR-545-3p are reduced. Changes in the plasma levels of these miRNAs suggest they could not only serve as early biomarkers for AD but also as potential therapeutic targets [86]. The above studies indicate that during drug development for neurodegenerative disorders such as AD and PD, in-depth investigation of the interactions between miRNAs and drug targets through high-throughput screening and bioinformatics tools can aid in identifying potential targets for new drugs and validating the efficacy of miRNAs as drug targets. This approach also allows for the optimization of existing drug mechanisms, enhancing their efficacy and specificity.

## CONCLUSION

With a deepening understanding of the ferroptosis mechanism, research is underway to develop ferroptosis inhibitors with higher selectivity, potency, and safety. For example, small molecule inhibitors and antibody drugs targeting key regulators of ferroptosis have attracted considerable attention. Additionally, gene editing and gene therapy technologies show promise in regulating ferroptosis [105], particularly integrating high-throughput screening and bioinformatics tools helps elucidate the interactions between miRNAs and drug targets. This approach not only aids in identifying new ferroptosis biomarkers and regulatory factors but also assists in discovering potential drug targets, thereby accelerating the discovery and development of new therapeutics.

However, research on ferroptosis in neural repair faces several challenges. Firstly, the mechanisms of ferroptosis are complex, involving multiple biological processes and signaling pathways, and its effects may vary across different types of neural cells. Secondly, ferroptosis often interacts with other forms of cell death, complicating both research and treatment efforts. Additionally, existing detection and quantification methods have limitations, necessitating the development of more sensitive and specific technologies. The development of regulatory factors also needs to be advanced for clinical application. Finally, the relationship between ferroptosis and neural repair remains incompletely understood, requiring further elucidation of its specific mechanisms. To address these challenges, it is recommended to enhance interdisciplinary collaboration, employ advanced technological approaches such as high-throughput screening and single-cell sequencing, conduct cell-specific studies, and develop novel detection tools. Additionally, integrating drug screening and comprehensive therapeutic strategies to explore ferroptosis modulation can advance its application and development in neural repair.

## Figures and Tables

**Fig. (2):**
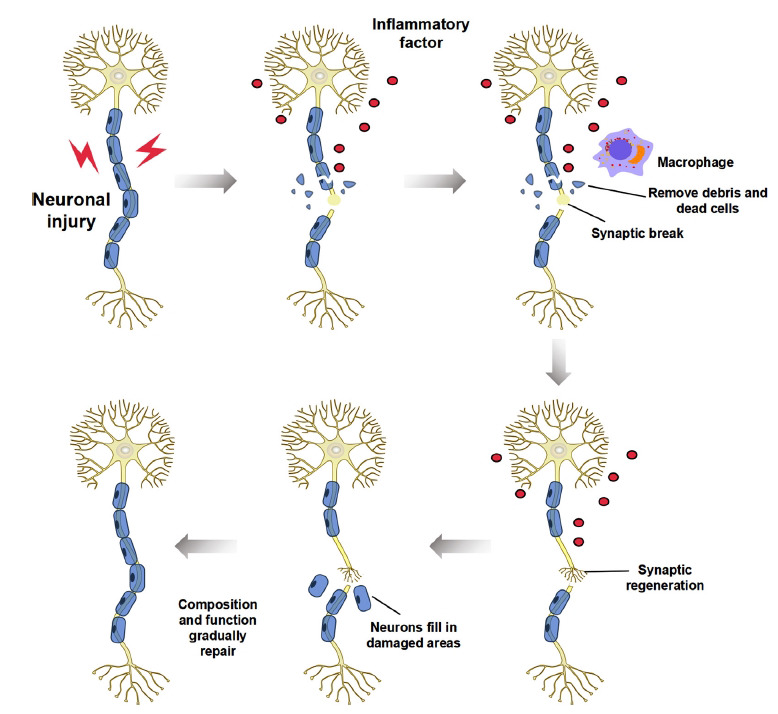
The processes of nerve repair.

**Table 1:** Natural plant products with potential.

**Type of Brain Injury**	**Natural Plant Products**	**References**
Alzheimer's disease	Isoforsythiaside	[87]
	Tetrahydroxy stilbene glycoside	[88]
	Eriodictyol	[89]
	Chalcone	[90]
	Hinokitiol	[91]
	Salidroside	[92]
Parkinson's disease	dl-3-n-butylphthalide	[93]
	Paeoniflorin	[94]
	Thoningianin A	[95]
	Hinokitiol	[96]
	Baicalein	[97]
	Puerarin	[98]
	Quercetin	[99]
Ischemic brain injury	Caffeic acid	[100]
	Rehmannioside A	[82]
	Luteolin	[101]
	Dihydroquercetin	[102]
	Icariin	[103]
	Matrine	[104]

## LIST OF ABBREVIATIONS

TF: Transferrin
TFR1: Transferrin receptor 1
STEAP3: Prostate 6 transmembrane protein 3
FPN: Ferroportin
LIP: Labile iron pool
DMT1: Divalent metal transporter 1
TBI: Traumatic brain injury
nTBI: Non-traumatic brain injury
ROS: Reactive oxygen species
DAMPs: Damage-associated molecular patterns
DFO: Desferrioxamine
DFP: Deferiprone
NAC: N-acetylcysteine
ALA: Α-lipoic acid
GPX4: Glutathione peroxidase 4
xCT: Cysteine/glutamate transporter
6-OHDA: 6-hydroxydopamine
N2L: Niacin dimer
TSG: Tetrahydroxystilbene glycosides
TCM: Traditional Chinese medicine
